# Atomic Interaction Networks in the Core of Protein Domains and Their Native Folds

**DOI:** 10.1371/journal.pone.0009391

**Published:** 2010-02-23

**Authors:** Venkataramanan Soundararajan, Rahul Raman, S. Raguram, V. Sasisekharan, Ram Sasisekharan

**Affiliations:** Harvard-MIT Division of Health Sciences & Technology, Koch Institute for Integrative Cancer Research and Department of Biological Engineering, Massachusetts Institute of Technology, Cambridge, Massachusetts, United States of America; Johns Hopkins School of Medicine, United States of America

## Abstract

Vastly divergent sequences populate a majority of protein folds. In the quest to identify features that are conserved within protein domains belonging to the same fold, we set out to examine the entire protein universe on a fold-by-fold basis. We report that the atomic interaction network in the solvent-unexposed core of protein domains are fold-conserved, extraordinary sequence divergence notwithstanding. Further, we find that this feature, termed protein core atomic interaction network (or PCAIN) is significantly distinguishable across different folds, thus appearing to be “signature” of a domain's native fold. As part of this study, we computed the PCAINs for 8698 representative protein domains from families across the 1018 known protein folds to construct our seed database and an automated framework was developed for PCAIN-based characterization of the protein fold universe. A test set of randomly selected domains that are not in the seed database was classified with over 97% accuracy, independent of sequence divergence. As an application of this novel fold signature, a PCAIN-based scoring scheme was developed for comparative (homology-based) structure prediction, with 1–2 angstroms (mean 1.61A) C_α_ RMSD generally observed between computed structures and reference crystal structures. Our results are consistent across the full spectrum of test domains including those from recent CASP experiments and most notably in the ‘twilight’ and ‘midnight’ zones wherein <30% and <10% target-template sequence identity prevails (mean twilight RMSD of 1.69A). We further demonstrate the utility of the PCAIN protocol to derive biological insight into protein structure-function relationships, by modeling the structure of the YopM effector novel E3 ligase (NEL) domain from plague-causative bacterium *Yersinia Pestis* and discussing its implications for host adaptive and innate immune modulation by the pathogen. Considering the several high-throughput, sequence-identity-independent applications demonstrated in this work, we suggest that the PCAIN is a fundamental fold feature that could be a valuable addition to the arsenal of protein modeling and analysis tools.

## Introduction

Nature employs merely a few thousand protein folds to generate the entire repertoire of the multimillion strong protein universe [1]. Massively divergent amino acid sequences thus populate protein families of many folds (Figure S1), ostensibly challenging the notion that all information dictating fold mapping of proteins—the protein fold code—is programmed in the sequence [2], [3]. We sought to decode conserved features within each fold family despite the vast degrees of sequence divergence, so as to better understand the factors governing the protein fold code. Given that the residues constituting the core are generally amongst the slowest evolving regions of protein structures [4] and are central to folding [5] and unfolding [6], we focused on the core of proteins to elucidate fold-conserved features.

**Figure 1:**
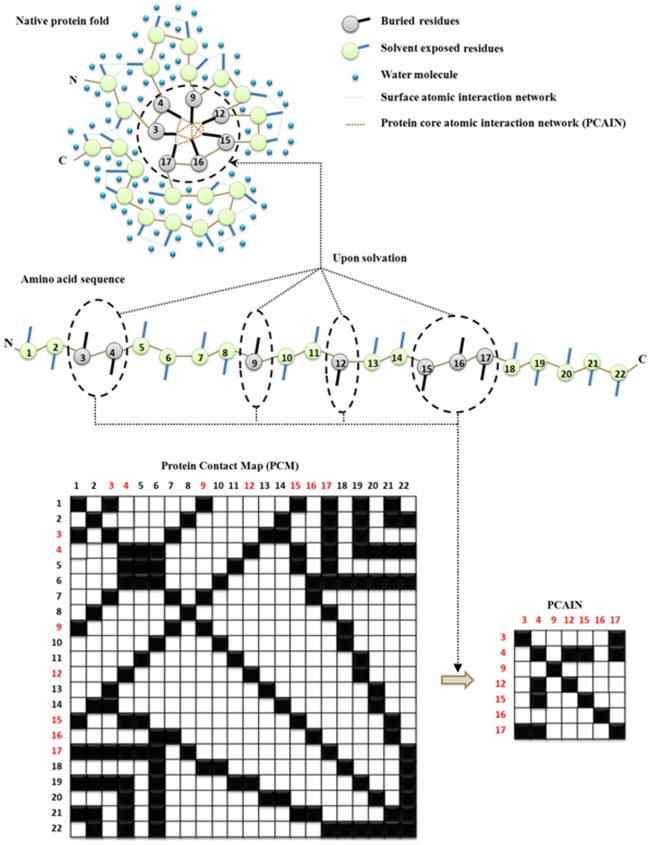
Computation of the protein core atomic interaction network (PCAIN) from the 2-D protein contact map (PCM). The PCM accounts for all atomic interactions in the 3-D protein structure while the PCAIN involves atomic interactions between just the conserved, solvent inaccessible residues in the ‘core’ of protein domains.

At the heart of a stable protein domain, are the solvent-unexposed residues in its core [7], [8]. The identity and packing of protein core residues are known to be key factors that mediate both the energetics of folding [9] and the emergence of fold families [10]. The quality of protein core packing has also proven useful to successfully refine and validate computationally generated structural models [11]. Recent studies have further examined specific families of proteins from sequence and packing/volume perspectives to delineate factors governing protein stability [12], [13]. Owing to the fact that atomic interactions are fundamental to defining protein folds, in this study, we considered the information content of protein contact maps (PCMs)—a function of the distance between atoms of all amino acids in a protein [14]. Further, in order to capture the information content in the solvent unexposed core regions of protein structures, we defined the *protein core atomic interaction network* or PCAIN (Figure 1). While different methods have been used to identify core residues of protein structures [7]–[14], we used conserved solvent inaccessibility as a metric to automate the identification of residues constituting the core of domains from protein family alignments (see Methods section) and focused exclusively on the atomic interactions between these residues to characterize each fold and compute a database of PCAINs.

We find that PCAINs are well-conserved between domains of the same fold family, while significantly different from the PCAINs for domains of other fold families—characteristics that are in sharp contrast to the non-fold-specific nature of PCMs. The fold-specific nature of PCAINs is further found to be consistent accross families from the entire universe of protein folds (numbering ∼1018), highlighting the PCAIN as “signature” of the native folded state of protein domains. Building on the fold-specific nature of PCAINs, we demonstrate the use of PCAIN-based scoring schemes for effective classification of protein sequences into their native folds and for high-throughput, accurate homology-based (comparative) protein structure prediction. We further highlight the potency of PCAINs for extending the current capabilities of homology modeling into the ‘twilight’ and ‘midnight’ zones [15], [16] of low target-template sequence identity (<30% and <10% respectively), including those from recent CASP experiments [17]. Having verified the utility of PCAINs, we proceed to estimate the sensitivity of PCAINs to threshold interaction distance (ρ) and conserved solvent accessibility (ω)—the two fundamental physical parameters that characterize the PCAIN—thus defining a (ρ, ω) landscape for protein structures. From this analysis, we find that the PCAIN is most refined around specific windows of (ρ, ω) values and propose an adaptive approach for maximizing the fold signature “signal” to evolutionary sequence divergence “noise”, thus enabling effective parameter-tuning of PCAINs for applications to derive biological insight into protein structure-function relationships. Finally, we showcase as an application of the developed protocols, PCAIN-based modeling of the hitherto unknown structure of the NEL domain from the YopM effector protein of plague-causative bacterium *Yersinia Pestis*. We conclude with discussions on the biological implications of the modeled bacterial protein structure, especially from the perspective of adaptive and innate immune signaling modulation during host-pathogen interplay.

## Results and Discussion

**Figure 2:**
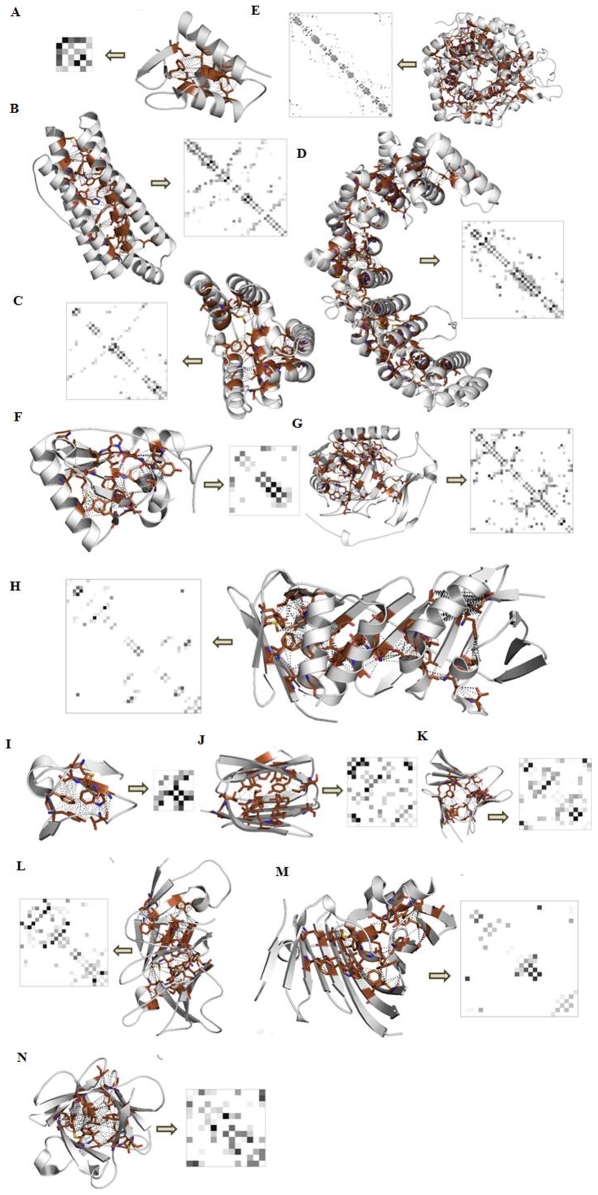
Snapshots from the PCAIN database used for mining fold-distinguishing signatures. The solvent inaccessible core of domains (shaded *brown*) from all 1018 naturally occurring folds were identified and used to compute the PCAINs (as described in the methods section) as part of the PCAIN database. Shown herein are representative domains and PCAINs (with *yellow* arrow between) from the following fold families–(A.) Orthogonal α-bundle (DNA helicase RuvA subunit); (B.) Up-down α-bundle (coiled-coil); (C.) α-horseshoe (leucine-rich repeat variant); (D.) α-solenoid (peridinin-chlorophyll protein); (E.) αα-barrell (glycosyltransferase); (F.) αβ-roll (HIV reverse transcriptase); (G.) αβ-complex (cytochrome); (H.) αβ-box (proliferating cell nuclear antigen); (I.) β-ribbon (seminal fluid protein PDC-109); (J.) β-sandwich (neurophysin); (K.) β-barrel (thrombin); (L.) β-propeller (pseudo β-propeller); (M.) β-clam (outer membrane lipoprotein receptor); (N.) β-trefoil (acidic fibroblast growth factor). Fold-distinguishing PCAIN patterns observed herein motivated systemic computation of intra-fold and inter-fold correlations on a family-by-family basis, as shown in supplementary figure S5. Fold-conserved interactions are evolutionary markers and are demarcated (*red* stars) on the corresponding sample set of the protein family alignments in supplementary figure S3.

We used the CATH database [18] as the source for our data on protein domains and their folds. At the time when this study was performed, the CATH database (Figure S2) had 112,450 protein domains classified into 1,018 folds. We chose 8,698 protein domains from accross the 1,018 folds representing all the different homologous superfamilies in CATH to seed our database. The structure-based multiple sequence alignments for the seeded domains were obtained from DHS [18] and conserved, solvent-unexposed core columns were identified for each alignment (Figure S3) using the solvent accessibility parameters from DSSP/CATH-wolf [19]–[21] for constructing the PCAIN database from the PCM database (Figure S4) as described in the methods section. As part of the PCAIN database, a comprehensive framework to document key conserved interactions for each family of the protein universe was developed (Figure 2), permitting assignment of PCAIN scores to threaded structures.

**Figure 3:**
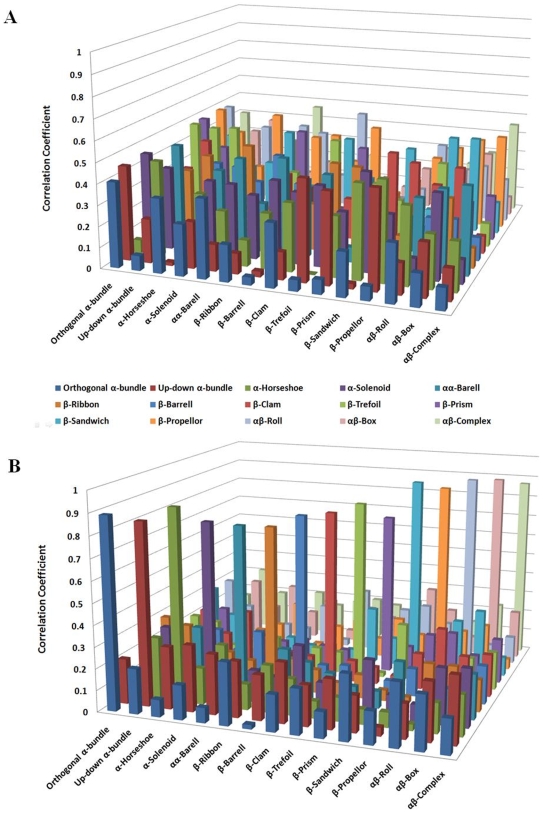
Contrasting the fold specificity of protein contact maps (PCMs) and protein core atomic interaction networks (PCAINs). Averaged intra-family (*diagonal*) and inter-family (*non-diagonal*) correlation coefficients of (A.) PCMs and (B.) PCAINs were computed at 5 angstroms threshold distance ρ and normalized solvent accessibility/atom of ω = 10 on a family-by-family basis for several prominent folds of the protein universe. The complete 1018 folds by 1018 folds correlations of PCMs and PCAINs for the entire fold universe is shown in supplementary figure S5. From these figures it is clear that PCAIN is highly fold-specific but PCM shows no discernible fold specificity.

In order to investigate the fold-specificity of PCAINs and contrast with that of PCMs, the averaged PCM and PCAIN scores for the seed domains from each of the 1018 folds were computed. The averaged PCM and PCAIN scores for all fold pairs were cross-correlated to obtain the correlation coefficients that provide for a quantitative estimate of variations in these scores for different folds (non-diagonal entries; Figure S5). The average degree of correlation in PCMs and PCAINs were also computed for each family, providing a quantitative estimate of the degree of fold-conservation for these scores (diagonal entries from top left to bottom right; Figure S5). From this data, it is clear that the PCM provides for no discernable fold-specificity owing to random correlations within (*diagonal)* and accross (*non-diagonal*) folds. On the other hand, it is evident that the PCAIN is highly fold-specific with low inter-family correlation coefficient values (*non-diagonal)* and high intra-family correlation coefficient values (*diagonal)*. In order to better illustrate this point, the PCM and PCAIN scores for several randomly selected fold families from architectures spanning a significant portion of the protein universe is also shown (Figure 3), from which the extremely high fold-specificity of PCAINs and low fold-specificity of PCMs is evident.

Given that the PCMs and PCAINs are functions of the threshold interaction distance (ρ) and conserved solvent accessibility (ω) parameters, the entire analysis was repeated for various threshold interaction distances ranging from ρ = 3.5–5.0 and conserved solvent accessibility cutoffs ranging from ω = 0–10, to observe consistently higher fold-specificity for PCAINs than PCMs (*data not shown)*. This analysis suggests that despite the large degree of sequence divergence in a majority of fold families, atomic interactions between amino acids in the solvent-unexposed core of domains (PCAINs) are a highly fold-conserved feature. The poor fold-specificity of the PCM on the other hand, is tell-tale of high “evolutionary tinkering” noise [22] drowning out the fold-conserved atomic interaction signals. Thus, it emerges that PCMs have high signal-to-noise (SNR) ratio and that the solvent accessibility parameter (ω) sieves out the function-driven evolutionary tinkering noise from PCMs. This implies that PCAINs are “de-noised filtrates” of PCMs - a result that corroborates the long-standing notion that exposure to solvent correlates with evolution-driven amino acid substitution [23]. Furthermore, from the perspective of 2-D and 3-D realms, this analysis suggests that solvent exposed atomic interactions are more liable to evolutionary tinkering than are solvent unexposed (buried) atomic interactions.

**Figure 4:**
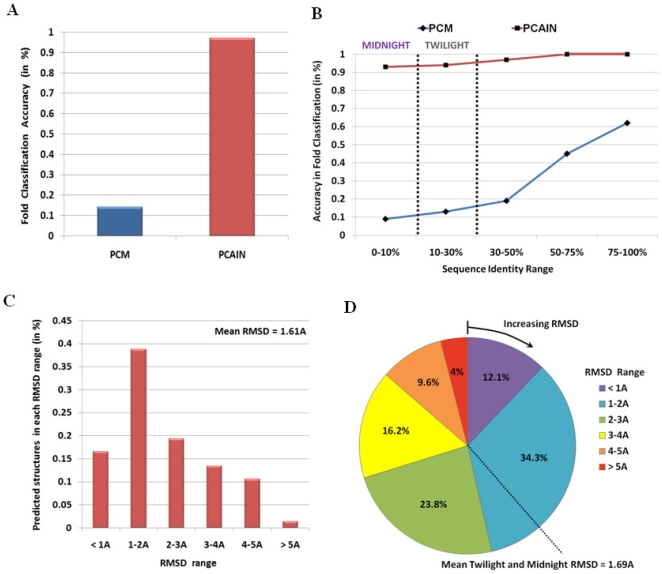
Applications of PCAIN as a divergence-independent metric for protein classification, anchored sequence alignment, and structure prediction. (A.) PCAINs were computed on a general screen of unselected protein domain sequences that were not part of the database and used to accurately classify these sequences as shown, confirming the fold-specific nature of PCAINs. PCMs of these domains are seen to be ineffective as classifiers in the general sequence space. (B.) PCAIN is seen to be an effective classifier regardless of the sequence identity of the target domain towards members of its native fold and is observed to be effective even in the twilight (<30% PSI) and midnight (<10% PSI) zones. On the other hand, the PCM is observed to be highly dependent on this sequence identity and provides for some moderate classification accuracy only in the high sequence identity range. (C.) The distribution of RMSD between PCAIN-based predicted structures and the reference crystal structures for target sequences with mean RMSD of 1.61A highlights the structure prediction efficacy of the proposed method. (D.) Pie chart of RMSD distribution for test sequences in the twilight and midnight zones is shown, indicating mean RMSD of 1.69A.

In order to examine the fold discriminating efficacy of PCAINs and PCMs with greater detail, a general screen of 50,000 randomly selected domains was considered from the universal set of 112,450 domains excluding the 8,698 representative domains from which the seed databases were constructed. While the PCAIN showed 97% accurate classification, the PCM showed only 14% accuracy in classification of domains into their respective folds (Figure 4A). Furthermore, the PCM's ability to classify folds was found to be heavily dependent on the target-template pairwise sequence identity (PSI), with an exponential decrease in classification accuracy with decrease in PSI (Figure 4B). It must be noted that in the higher PSI realm (>50%) wherein the PCM shows some marginal performance, sequence-based (1-D) methods are known to perform significantly well [24] and the utility of the 2-D PCM based approach is defeated owing to the higher computational cost involved. On the other hand, the PCAIN is found to be largely uninfluenced by the drop in PSI and consistently shows over 95% fold-classification accuracy even in the twilight (<30% PSI) and midnight zones (<10% PSI) (Figure 4B). This analysis showcases the 2-D PCAIN as a useful tool to add to the existing methods for protein fold recognition such as profile pattern recognition and protein threading [25]–[28].

While some existing methods are able to recognize folds accurately [25]–[27], there is still an unmet need for methods that can proceed from fold recognition towards accurate *homology-based structure prediction* [28] in the ‘twilight’ and ‘midnight’ zones—wherein target-template sequence identity are <30% and <10% respectively [15], [16]. Furthermore, this breakdown of homology modeling utility with low target-template identity challenges elucidation of structures for newly discovered proteins, several of which happen to fall into the twilight and midnight zones [26], [29], [30]. To address this issue, we systematically evaluated the potency of the PCAIN approach for homology-based structure prediction, motivated by the high fold-specificity of PCAINs. For this purpose, we developed a PCAIN-based scoring scheme (Figure S6) outlined in the methods section—for template selection, anchored sequence alignment, and homology-based structure prediction. This testing was performed with a general screen of randomly selected domains from the universal set of domains, excluding the representative domains of the seed database, and including those from recent CASP experiments.

The reference structure-based sequence alignments were seen to have extremely high correlations to the PCAIN-based anchored alignments with pearson's correlation coefficient of 0.91. It is interesting that atomic interactions are mined from 3-D structural coordinates and 2-D PCAINs are used to identify the fold-conserved set of atomic interactions that are finally mapped to thread 1-D amino acid sequences. This underlines the application of fold-conserved (including in twilight and midnight zones) higher dimensional data from structural (3D) and contact (2D) spaces for effective protein analysis. This also establishes that PCAIN-based anchored alignments closely mimic the actual structure-based sequence alignments, thus confirming the utility of PCAINs vis-a-vis sequence alignment. Furthermore, superposition of the modeled test structures onto the reference crystal structures demonstrated good structure prediction accuracy in the range of 1–2 angstroms, with mean RMSD of 1.61 angstroms (Figure 4C). In order to specifically estimate the efficacy of the PCAIN approach for structure prediction in the twilight and midnight zones of sequence identity, the RMSD range for the predicted structures corresponding to the test domains in these zones was also computed (Figure 4D). The mean RMSD in the twilight and midnight zone was 1.69 angstroms with the overall RMSD distribution (Figure 4D) very similar to that obtained for the entire set of test domains (Figure 4C), thus proving that the PCAIN approach to structure prediction is sequence-identity-independent and hence notably potent in twilight-midnight zones. Successful prediction of structures for example targets from recent CASP (critical assessment of structure prediction) proceedings that are not part of the CATH database or the seed datasets further illustrate the generic, database-independent efficacy of the PCAIN approach (Figure S7). This analysis confirms the high-throughput accuracy of PCAIN-based structure prediction and showcases it as a valuable addition to the arsenal of structural modeling tools.

The significantly improved performance of PCAINs over PCMs [31] is due to three distinct advantages. Primarily, owing to de-noising of “evolutionary tinkered” contacts from the PCM, the PCAIN enables exclusive retention of fold-specific signals. Next, the PCAIN scores for sequences generally best match with the representative domains from the same superfamily, rather than domains of other superfamilies even belonging to the same fold. Since protein folds are classified into superfamilies based on common functions and evolutionary relationships, it is likely that the PCAIN-based methodology enables handpicking of an optimal functionally-related template molecule for modeling the structure of the unknown protein, thus contributing significantly towards improving the accuracy of structure prediction. Finally, the PCAIN methodology provides for utilizing the fold-conserved residues as “anchors” in the target-template sequence alignment step, thus increasing efficacy of conventional alignment protocols. Taken together, these three factors contribute towards the potency of PCAINs for the discussed applications. With further improvements to the accuracy of secondary structure prediction methods and incorporation of additional fold-conserved features from solvent-exposed regions, it is conceivable that more accurate structures may be predicted as part of future advancements to the PCAIN methodology.

**Figure 5:**
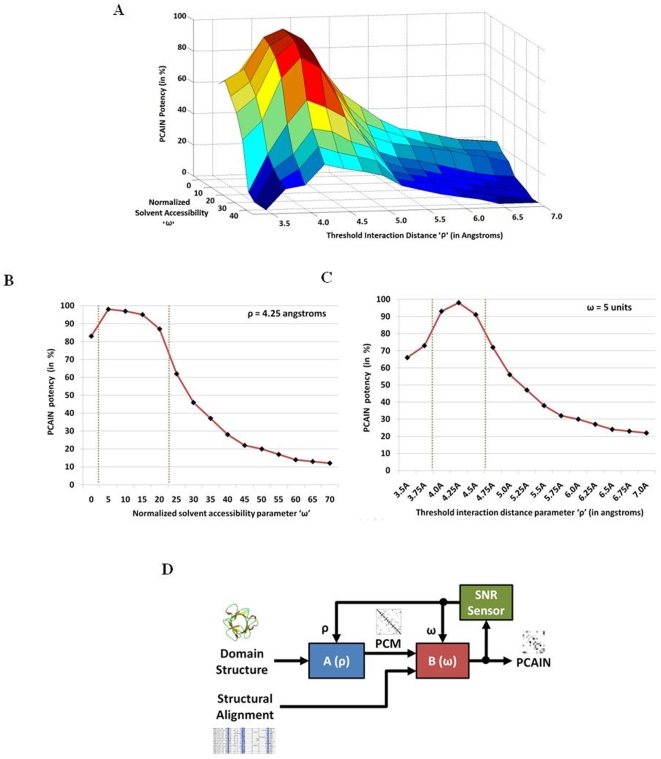
PCAIN as a function of threshold interaction distance (ρ) and conserved solvent accessibility (ω) parameters. (A.) Variation of PCAIN potency (difference between averaged intra-fold and inter-fold PCAIN correlations) with threshold interaction distance ρ and conserved solvent accessibility ω. (B.) At fixed ρ = 4.25 angstroms, the variation of PCAIN potency with ω. (C.) At fixed ω = 25, the variation of PCAIN potency with ρ. (D.) Implementation of adaptive tuning of ρ and ω parameters for maximizing SNR.

Given that the PCAIN is a function of two fundamental parameters, namely, threshold interaction distance (ρ) and conserved solvent accessibility (ω), we investigated the effect of modulating these parameters (Figure 5). For this purpose, a parameter scan on (ρ, ω) was performed and the effective operable landscape for PCAIN-based methods was mapped for the range ρ = 3.5–7.0 angstroms and ω = 0–40%. Given that high intra-family PCAIN correlation scores and low inter-family PCAIN correlation scores are necessary for defining a refined fold signature with high SNR, the difference between these two scores provides a reliable measure of potency. We find that the PCAIN is sensitive to both the threshold interaction distance parameter (ρ) and the conserved solvent accessibility parameter (ω), with higher sensitivity towards the former (Figure 5A). Specifically, the PCAIN is found to be most effective as a fold signature (high intra-family and low inter-family correlations) in the window ω = 2–20% (Figure 5B) and similarly in the window ρ = 4.0–4.5 angstroms (Figure 5C).

The (ρ,ω) landscape may be interpreted as follows. Protein structures are ensembles of backbone bonded dipeptide confirmations that are characterized by the (ϕ, ψ) plot [32]–[34] and other side-chain interactions that are characterized by inter-residue distance [35]. Too much threshold interaction distance (ρ) implies accounting for non-influential residue pairs as interactions and such *pseudo-interactions* will add to the noise thus decreasing SNR and PCAIN potency. Too little threshold interaction distance (ρ), on the other hand, is not feasible, since it will be less than inter-atomic Van der Waals distances. The ‘ω’ parameter accounts for the interplay between water molecules and the residues constituting the protein structure and from this perspective the PCAIN may be viewed as essentially the solvent unexposed network (SUN) of interacting residues. Specifically, a higher ‘ω’ value implies accounting for partially solvent exposed (and hence possibly non-conserved) atomic interaction networks, thus adding to the noise factor and decreasing PCAIN effectiveness. A ‘ω’ value close to zero, on the other hand, may be too stringent. Along the lines of this analysis, it is conceivable that fine-tuning of the PCAIN may be required for specific molecular biology applications. Having mapped the effective operable landscape for PCAIN-based methods with the goal of obtaining the maximal PCAIN effectiveness and highest possible SNR, we propose an adaptive framework (Figure 5D) for such fine-tuning of the (ρ, ω) parameters as required by the application of interest.

Protein fold recognition and structure prediction have numerous biological applications [28]–[30]. In addition to the previously demonstrated applications of sequence alignment, fold identification, template selection, and homology modeling, we demonstrate herein, application of the described PCAIN-based structure prediction methodology to derive biological insight into potential structure-function relationships of proteins with hitherto unresolved structure. As an example to highlight this application, we consider the effector protein YopM from the plague-causative bacterium *Yersinia pestis* [36]. While it is well-known that YopM is a critical virulence determining factor, structural insight into potential roles of YopM in *Y. pestis* pathogenesis has been elusive, due to the unsolved structure of the YopM novel E3 ligase (NEL) domain [37].

**Figure 6:**
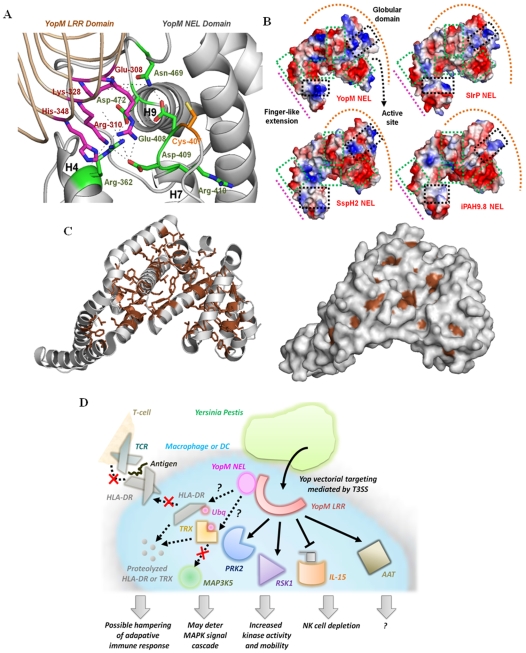
Application of the PCAIN methodology to analyze potential structure-function relationships of the novel E3 ligase (NEL) domain from the YopM effector protein of the plague-causative bacterium *Yersinia Pestis*. (A.) The YopM NEL domain structure was modeled using the PCAIN methodology and the putative ubiquitin ligase catalytic site was characterized, based on the recent experimental characterizations of *Salmonella* and *Shigella* NEL domains [38]–[41]. The likely hydrogen bonds that stabilize the active site (*black* lines) and the key α-helices (H4, H7, and H9) are indicated. (B.) Vacuum electrostatics of the molecular surfaces from superposed NEL domains of YopM, SlrP, SspH2, and ipaH were generated (*see* *Methods*) with negative, positive, and neutral patches colored *red*, *blue*, and *white* respectively. The finger-like extension (*pink* line), globular domain (*orange* arc), and active site location (*black* arrow) are indicated. (C.) The solvent-unexposed residues that constitute the PCAIN of the modeled YopM NEL domain structure (*gray*) are shown as sticks (*brown*). The molecular surface of the YopM NEL domain is also shown alongside to highlight that the residues constituting the PCAIN (*brown*) are only very minimally solvent exposed. (D.) This is a pictorial depiction of YopM in the intracellular context and the key structural implications for its modulation of human adaptive and innate immune signaling. Specifically, YopM is known to interact with protein kinase C-like 2 (PRK2) and ribosomal S6 protein kinase 1 (RSK1) resulting in increased activity and mobility of these kinases, in addition to potentiating natural killer (NK) cell depletion by suppressing expression of Interleukin-15 (IL-15) [37]. YopM has also been shown to specifically interact with α1-antitrypsin (AAT) without affecting its anti-protease activity, due to which the biological significance of this interaction remains unknown.[37] Also indicated by the question mark (?) symbols are hitherto unknown interactions for YopM, extrapolated based on the functions of the related proteins. Specifically highlighted in this regard are the degradation of human leukocyte antigen-DR (HLA-DR) and thioredoxin (TRX) that may cause suppression of adaptive immune response via moderation of antigen presentation and modulation of innate immune signaling via the MAPK cascade, respectively. It remains to be seen what precise intracellular molecules are targeted by YopM NEL for proteolytic degradation, considering the autoregulated ubiquitin ligase activity suggested by our PCAIN-based model and analysis.

We modeled the YopM NEL domain structure using the PCAIN methodology and investigated the putative ubiquitin ligase catalytic site (Figure 6A). From the modeled structure, we note remarkable correlation in molecular surface electrostatics including the highly-conserved patches (Figure 6B), in NEL domain structures from *Salmonella* SspH2 [38], *Salmonella* SlrP [39], *Shigella* IpaH [40], and *Yersinia pestis* YopM, in addition to high correlation of the PCAINs for these domains (Figure 6C ). Given that these patches constitute the NEL catalytic site [40] and the recently characterized *Salmonella* NEL domain interaction sites with human leukocyte antigen-DR (HLA-DR; a major histocompatibility complex (MHC) class II receptor) and thioredoxin (TRX) [38], [39], it is likely that the YopM NEL domain functions as an autoregulated E3 ubiquitin ligase and degrades human intracellular proteins, similar to NEL domains from *Salmonella* and *Shigella*. Such an ubiquitinase activity of YopM NEL has significant implications for modulation of host adaptive and innate immune response to plague (Figure 6D). The ubiquitination and subsequent degradation of HLA-DR by *Salmonella* effectors within antigen presenting cells like macrophages, B-cells, and dendritic cells, has been recently shown to diminish the surface expression of MHC class II antigens [41]. It is conceivable that a similar interaction of YopM NEL with HLA-DR could moderate the host adaptive immune response (Figure 6D). Confirmation of the proteolytic degradation of TRX by YopM will have important implications in the regulation of mitogen-activated protein kinase kinase kinase 5 (MAP3K5) signaling, for TRX interaction with MAP3K5 [42] provides *Y.pestis* a plausible direct method to modulate innate immunity (Figure 6D). More specifically, future studies that biochemically characterize interactions of key host intracellular molecules to the YopM molecule modeled herein, will further our understanding of the specific mechanisms governing bacterial subversion of human adaptive and innate immune signaling pathways.

The modeling of YopM NEL domain demonstrated in this study amply highlights application of the PCAIN methodology to derive biological insight into protein structure-function relationships. Taken together with the previously described applications of the PCAIN methodology such as sequence alignment, fold identification, template selection, and structural modeling, our study confirms the PCAIN as a fundamental fold feature that will be a valuable addition to the arsenal of protein modeling and analysis tools. Additionally, the PCAINs computed as part of this work (such as those from the database shown in Figure 2) are likely to be useful resource for molecular engineering applications since they provide a rigorous starting framework or *scaffold* upon which rest of the protein design may be tailored based on the functions of interest. PCAIN computation and analysis may also be valuable for applications such as elucidating mechanisms of protein evolution, stability, folding, unfolding, and misfolding, given the central role of the protein core in governing these phenomena [43]–[46].

It has recently been shown that two specific amino acid sequences with overwhelming identity (∼88%) adopt distinct folds, thus postulating that for the specific protein pair considered, only ∼12% of the amino acid sequence codes for sequence-to-structure mapping [47]. PCAIN sheds light on a “*fold code*” that is consistently encoded into residues that constitute the networks of atomic interactions in solvent unexposed core regions of protein native structures. This suggests that the fold code is a *network phenomenon* along with sequence and structural phenomena, thus providing rationale as to why merely sequence-based or structure-based pattern analysis of proteins may not succeed in decoding fold signatures. The cores of the protein domains of the same fold as identified by our method can have low sequence identity and poor secondary structure motif matching, but high conservation of their PCAINs (Figure S8). Hence, defining protein cores based on treatment of protein structures as atomic networks characterized by the (ρ, ω) plot and denoising of PCMs by recognition of signature network patterns, distinguishes our PCAIN methodology from the previously explored knowledge-based threading potentials. Our finding that the atomic interactions between just 15–20% of residues in native structures of each examined fold are conserved, further suggests that the PCAIN is a minimalistic fold code.

Finally, this study provides compelling evidence in support of Anfinsen's dogma [48] that information dictating the native structural fold of protein domains is encoded in its amino acid sequence. Herein we have shown that “a significant portion of the fold-dictating information is encoded by the atomic interaction network in the solvent-unexposed core of protein domains”.

## Materials and Methods

### Automated Identification of ‘Core’ Residues and Construction of a Core Composition Database Characterizing All 1018 folds of the Protein Universe

At the time when this study was performed, the CATH database [18] had 112,450 protein domains classified into 1,018 folds, from which 8,698 protein domains representing the different homologous super families were used to seed our database. CATH defines cores based on secondary structural element analysis, whereas in our method the core can include non-secondary structural elements. Taken together with several other methodology distinctions, the cores identified by us are unique (as highlighted for the illustrative domain in Figure S8 for which more than 75% of CATH and PCAIN core residues are distinct). The structure-based multiple sequence alignments were obtained from DHS [18] (Figure S3) and the absolute solvent accessibility (ASA) factors from DSSP/CATH-wolf [21] were obtained for the amino acids of all 8,698 domains. The relative solvent accessibility (RSA) per atom was computed for each residue. The mean solvent accessibility (ω) was then calculated for all columns of the seed alignments and a threshold was used to identify the consistently solvent-unexposed columns as shown (Figure S3). This set of consistently solvent inaccessible columns was mapped back onto the conserved residue positions thus defining the core for all the seeded protein domains from each alignment. This was compiled into a dataset of protein core residues, one corresponding to each protein family and each considered value of parameter ω. The frequency of each amino acid at the core positions was also consolidated into a dataset of family-specific protein core residue propensities. The complete protein core characterization method, right from CATH mining until the construction of the datasets was automated with the implementation of a script in MATLAB 7.6.0 from The MathWorks, Inc. (Nattick, MA).

### Automated Construction of the PCM and PCAIN Databases for All 1018 Folds of the Protein Universe

A MATLAB script was written to automate the computation of protein contact maps (PCMs) for all seeded domains of the 1018 folds at various threshold interaction distance parameter (ρ) values (Figure S4). This was compiled into a database of PCMs on a fold-by-fold basis. The previously identified core residues for each domain of each fold at various ω values was used to identify the rows and columns of interest from PCMs at various ρ values and these were concatenated into the corresponding PCAINs for each domain of each fold at various (ρ, w) values, as depicted pictorially (Figure 1). This step was automated with a MATLAB script, which was also ultimately used to compile the generated PCAINs into an integrated PCM-PCAIN database for various (ρ, w) values. A simple python script was written and executed in PyMol for visualization of all the protein cores and PCAINs shown in this study (Figure 2). The pearson's correlation coefficient was computed to quantitatively contrast PCMs and PCAINs both within and accross all 1018 folds (Figure S5) and accross 15 unselected folds for refined visualization purposes (Figure 3).

### Automated Fold Classification of Randomly Selected Domains from the Protein Universe

(Figure S6)–A general screen of 50,000 randomly selected domains (obtained from the set of 112,450 domains excluding the 8,698 representative domains in the training set from which the PCM and PCAIN databases were constructed) were considered for testing the fold classification efficacy of PCAIN-based and PCM-based scoring schemes. The effectiveness of the classification approaches were then estimated (Figure 4A) using the actual folds of the test sequences as reference. Variations of the classification efficacies as a function of target-template sequence identity were also computed (Figure 4B).

### Template Selection Based on Target PCAIN Estimation and Correlation with Protein Family PCAIN Signatures

An automated MATLAB script was written to compute the secondary structures of the target amino acid sequences based on secondary structure prediction consensus [49]–[51]. The type, quantity and distribution of secondary structures are partially characteristic of folds and offer a good first filter for the fold and template selection process. Potential amino acids that correlate with the propensity data for each core position of all the screened folds are then identified for the target sequences, providing an estimate of ‘core fit’ and serving as a second filter for fold and template selection. The algorithm for this step is also implemented in MATLAB 7.6.0 from The MathWorks, Inc (Natick, MA) and accepts three inputs, namely, target amino acid sequences, the corresponding secondary structural information, and the fold-specific core residue propensity dataset. The target sequences for which all potential core residues are identifiable are deemed ‘core fit’ with respect to the screened folds and the target PCAIN scores for these are computed using the PCAIN database. For a majority of cases, the identical residue pairs are present in the database and hence their corresponding pairwise score is directly utilized. In other cases, an average of pairwise interactions between the two considered core positions from all other members of the screened fold family is used in this step. The target PCAIN scores are subsequently back-correlated with the averaged PCAIN score of each family and the resulting correlation coefficients provide an additional estimate of the degree of ‘core fit’. A simple threshold step is used at this stage as the third and final filter to determine the protein family, thus providing for selection of the optimal template molecule.

### Automated Anchored Sequence Alignment and Comparative Structure Prediction for Randomly Selected Protein Domains

The steps of this algorithm are depicted as a flowchart in Figure S6. Briefly, a general screen of randomly selected domains were obtained from the set of 112,450 domains (excluding the 8,698 representative domains for which PCM and PCAIN databases were constructed) and their PCAINs were estimated as detailed above. The computed target PCAIN scores were then correlated with the PCAIN scores (from the seed database) of every representative homologous superfamily member of the identified fold family in order to compute the optimal template, based on similarities at the level of evolutionary origin and function. The corresponding scaffold residues of the target and template sequences are then ‘anchored’ and pairwise sequence fragments between subsequent anchors are aligned using standard functions from the MATLAB bioinformatics toolbox with the BLOSUM62 scoring matrix and default gap penalties. The process involving fold identification, template selection and anchored alignment is maximally automated with the design of a MATLAB-based model. The structure-based sequence alignments are correlated with the PCAIN-based anchored alignments to estimate the efficacy of the PCAIN approach to sequence alignment (Figure 4C). Once the optimal anchored target-template alignments were computed, these were input to the automated homology modeling script of Discovery Studio from Accelrys, Inc. (San Diego, CA) that uses standard force fields to determine the energy minimized 3-D structural coordinates for the test sequences, including those from recent CASP experiments (as illustrated by examples in Figure S7). Each modeled 3D structure was then superposed onto the actual crystal structure obtained from the PDB using an automated MATLAB function and the root mean square deviations upon superposition were computed (Figure 4D).

### Modeling NEL Domain Structures with the PCAIN Methodology and Analysis of Their Putative Structure-Function Relationships

The molecular structures of NEL domains from *Yersinia pestis* YopM (NCBI Reference Sequence: ZP_02316950.1) and *Salmonella typhimurium* SlrP (GenBank: AAD39928.1) were modeled using the described PCAIN methodology with the identified optimal template structure of *Shigella* type III effector IpaH (PDB ID: 3CKD). All structure-function relationship analysis, including vacuum electrostatics generation for the modeled *Yersinia pestis* YopM NEL, modeled *Salmonella typhimurium* SlrP NEL, crystal structures from *Shigella* IpaH NEL (PDB ID: 3CKD), and *salmonella* SspH2 NEL (PDB ID: 3G06), were performed with PyMol.

## Supporting Information

Figure S1 Evolutionary sequence divergence of protein families. More than 60% of protein families from the pfam database were found to be significantly divergent in their sequences (High range), around 30% of protein families were found to be moderately divergenct in ther sequences (Medium range) and less than 10% of protein families were found to be well conserved in their sequences (Low range). This shows that evolutionary tinkering and sequence divergence are rampant across the protein universe.
(0.14 MB JPG)

Figure S2 The diversity of protein folds. Representative protein domains from CATH showcasing the fold diversity, classified according to their class (mainly α/mainly β/αβ) and architecture.
(0.11 MB JPG)

Figure S3 Sample sets from fold family alignments highlighting the solvent-unexposed (core) conserved positions (blue columns). (A) Sample proteins from a family of the architecture - Orthogonal bundle. (B) Sample proteins from a family of the architecture - Up-down bundle. (C) Sample proteins from a family of the architecture - Alpha-horseshoe. (D) Sample proteins from a family of the architecture - Alpha-alpha Barrel. (E) Sample proteins from a family of the architecture - Beta-Ribbon. (F) Sample proteins from a family of the architecture - Beta-Barrel. (G) Sample proteins from a family of the architecture - Beta-Trefoil. (H) Sample proteins from a family of the architecture - Beta-Prism. (I) Sample proteins from a family of the architecture - Beta-Sandwich. (J) Sample proteins from a family of architecture - Beta-Propeller. (K) Sample proteins from a family of architecture - αβ Roll. (L) Sample proteins from a family of architecture - αβ Box. (M) Sample proteins from a family of architecture - αβ Complex.
(1.50 MB JPG)

Figure S4 A sample dataset from the protein contact maps (PCM) database. The inter-residue contact maps at 5 angstroms threshold distance are shown for representative domains from a diverse set of topologies/folds spanning all natural architectures in the protein universe.
(0.19 MB JPG)

Figure S5 Protein contact maps (PCMs) versus protein core atomic interaction networks (PCAINs) intra- and inter- fold family correlations reveals striking specificity for PCAIN across the universe of folds. Averaged intra-fold (diagonal) and inter-fold (non-diagonal) correlation coefficients of (a.) PCMs and (b.) PCAINs at 5 angstroms threshold, shows clears that the PCAIN is highly fold-specific whereas the PCM shows no discernible fold specificity.
(0.43 MB JPG)

Figure S6 Flowchart governing PCAIN-based fold recognition of target sequence, template selection, anchored target-template alignment, and homology-based structure prediction. The detailed procedures associated with each step are described in the methods section. Briefly, a combination of secondary structure distribution and PCAIN scores from the key interaction positions was used to (i.) identify the fold of the target sequence, (ii.) compute the ideal template structure based on the closest functional homolog estimated from the superfamilies of the identified fold, (iii.) converge on the set of ‘anchor’ positions between the target and template sequences based on protein core amino acid frequencies to compute the optimal anchored target-template alignments, and (iv.) determine the target domain's 3-D structural coordinates from the anchored alignments with an automated homology modeling script.
(0.05 MB JPG)

Figure S7 Superposition of structures predicted based on PCAIN methodology for CASP (Critical Assessment of Structure Prediction) target sequences (a.) TO203 and (b.) TO197, illustrates PCAIN-based structure prediction. PCAIN-based structures predicted (cyan) are superposed onto reference crystal structures (pink) for (a.) TOP203 and (b.) TO197 from CASP-6 with RMSDs of 0.91A (at 29% target-template sequence identity) and 0.87A (at 60% target-template sequence identity) respectively. The corresponding results of structure prediction accuracy from the CASP models shown as tables shows minimum RMSDs of 1.29A and 1.37A respectively.
(0.26 MB JPG)

Figure S8 Defining protein cores and extracting their information with the PCAIN methodology. (A.) Polar and charged residues (yellow) are also part of the core of protein domain as identified by our method, as shown with E.coli thioredoxin (cyan) as an example. (B.) Only 7% identity (shaded green) is present in the sequence of residues that constitute the core of glutaredoxin and thioredoxin that adopt the same fold, whereas 93% of the core residues are different in identity (shaded yellow). However, the PCAINs of these two proteins are seen to have 98% correlation, over the PCMs that have only 41% correlation. This example further illustrates that the identity or hydrophobicity of residues are poor tools for extracting information from protein cores, whereas the PCAIN is optimal for extracting conserved information from protein cores. Similarly, very poor overlap is seen between residues used for CATH alignments (underlined) and the residues that contribute to the PCAIN, thus illustrating the novelty in determination of PCAIN residues.
(0.07 MB JPG)
