# Supplementary figures and images for: Atomic Interaction Networks in the Core of Protein Domains and Their Native Folds
Source: PLoS One. 2010 Feb 23;5(2):e9391. doi: 10.1371/journal.pone.0009391 (PMC2826414; doi:10.1371/journal.pone.0009391)

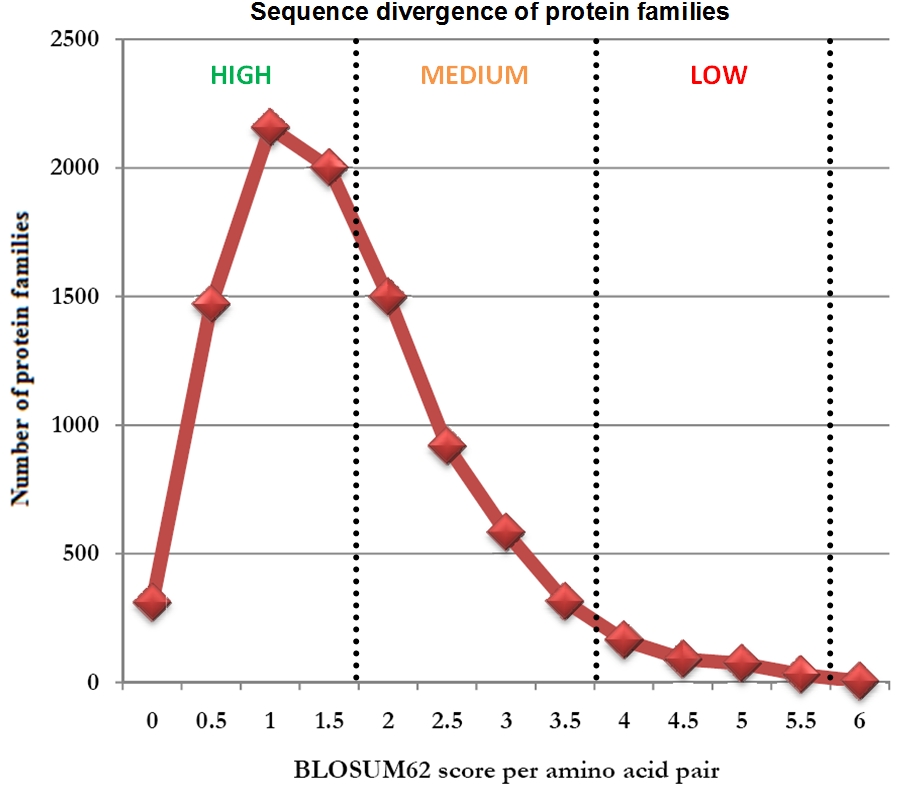

Supplement: Figure S1 — Evolutionary sequence divergence of protein families. More than 60% of protein families from the pfam database were found to be significantly divergent in their sequences (High range), around 30% of protein families were found to be moderately divergenct in ther sequences (Medium range) and less than 10% of protein families were found to be well conserved in their sequences (Low range). This shows that evolutionary tinkering and sequence divergence are rampant across the protein universe. (0.14 MB JPG) [file pone.0009391.s001.jpg]

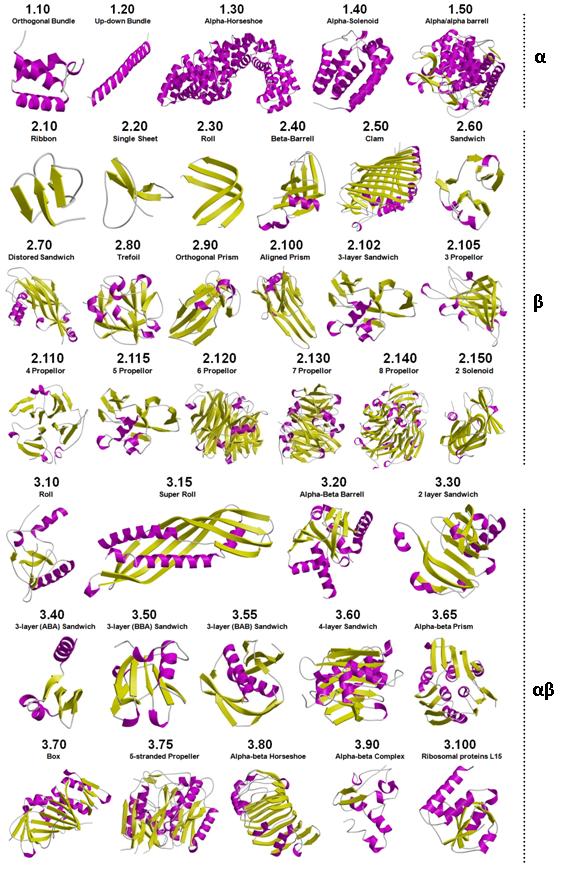

Supplement: Figure S2 — The diversity of protein folds. Representative protein domains from CATH showcasing the fold diversity, classified according to their class (mainly α/mainly β/αβ) and architecture. (0.11 MB JPG) [file pone.0009391.s002.jpg]

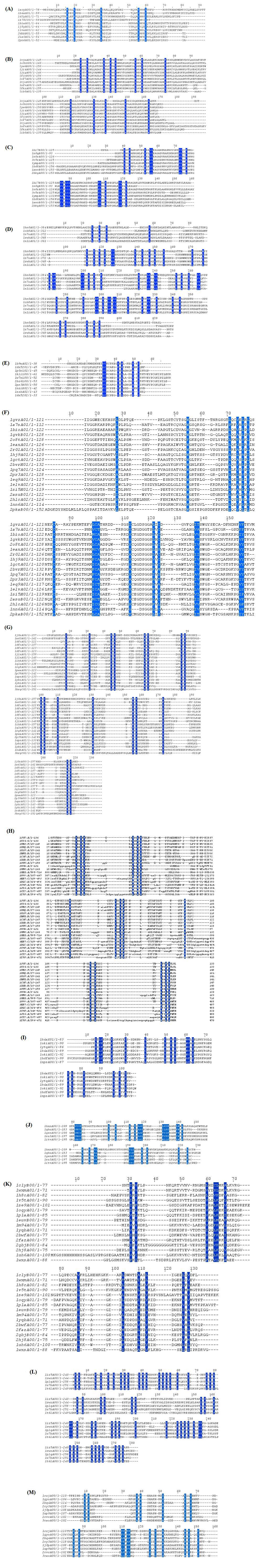

Supplement: Figure S3 — Sample sets from fold family alignments highlighting the solvent-unexposed (core) conserved positions (blue columns). (A) Sample proteins from a family of the architecture - Orthogonal bundle. (B) Sample proteins from a family of the architecture - Up-down bundle. (C) Sample proteins from a family of the architecture - Alpha-horseshoe. (D) Sample proteins from a family of the architecture - Alpha-alpha Barrel. (E) Sample proteins from a family of the architecture - Beta-Ribbon. (F) Sample proteins from a family of the architecture - Beta-Barrel. (G) Sample proteins from a family of the architecture - Beta-Trefoil. (H) Sample proteins from a family of the architecture - Beta-Prism. (I) Sample proteins from a family of the architecture - Beta-Sandwich. (J) Sample proteins from a family of architecture - Beta-Propeller. (K) Sample proteins from a family of architecture - αβ Roll. (L) Sample proteins from a family of architecture - αβ Box. (M) Sample proteins from a family of architecture - αβ Complex. (1.50 MB JPG) [file pone.0009391.s003.jpg]

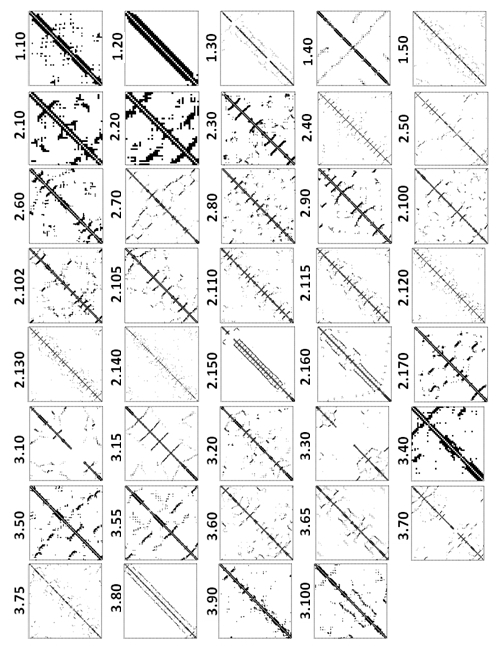

Supplement: Figure S4 — A sample dataset from the protein contact maps (PCM) database. The inter-residue contact maps at 5 angstroms threshold distance are shown for representative domains from a diverse set of topologies/folds spanning all natural architectures in the protein universe. (0.19 MB JPG) [file pone.0009391.s004.jpg]

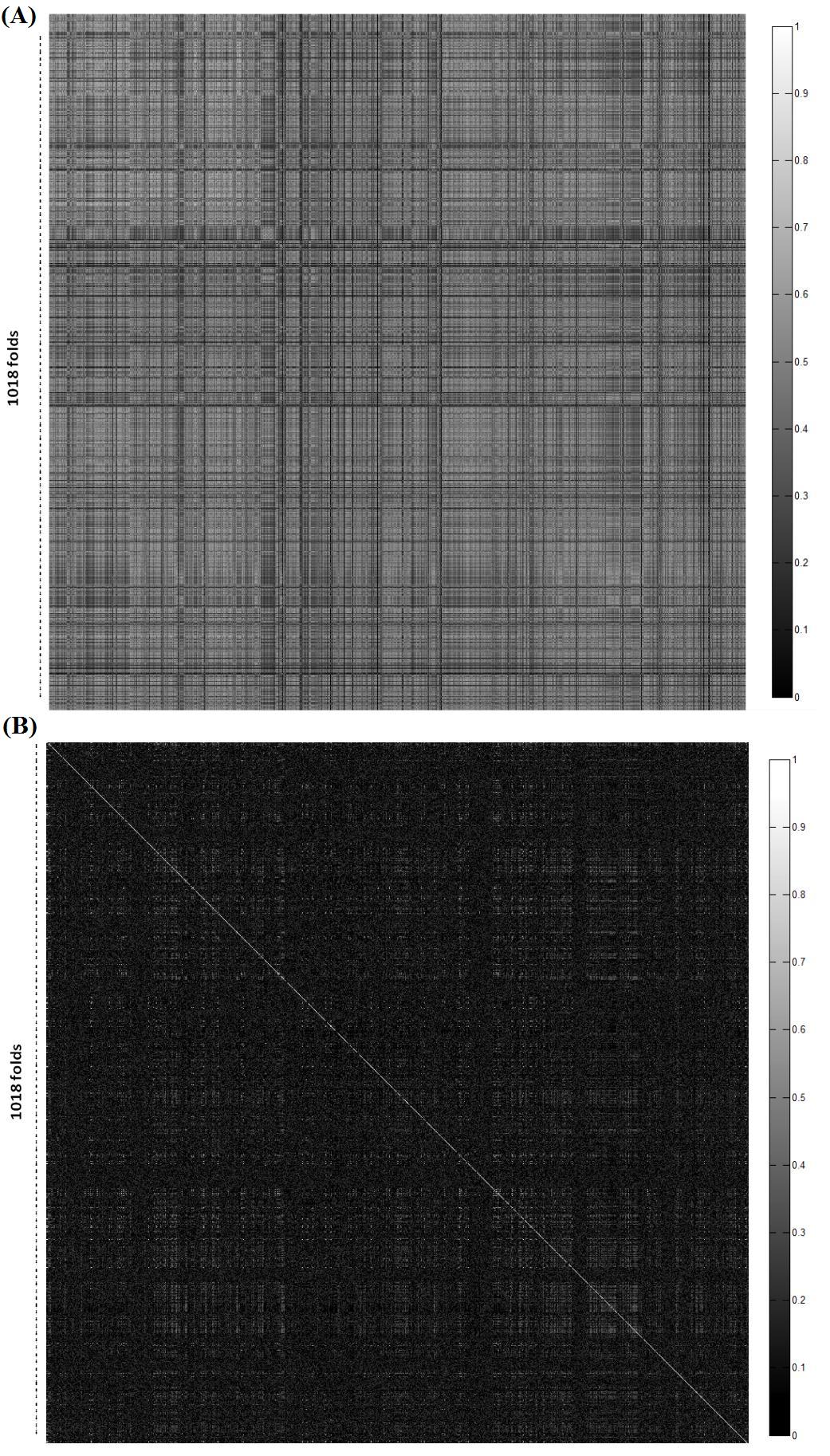

Supplement: Figure S5 — Protein contact maps (PCMs) versus protein core atomic interaction networks (PCAINs) intra- and inter- fold family correlations reveals striking specificity for PCAIN across the universe of folds. Averaged intra-fold (diagonal) and inter-fold (non-diagonal) correlation coefficients of (a.) PCMs and (b.) PCAINs at 5 angstroms threshold, shows clears that the PCAIN is highly fold-specific whereas the PCM shows no discernible fold specificity. (0.43 MB JPG) [file pone.0009391.s005.jpg]

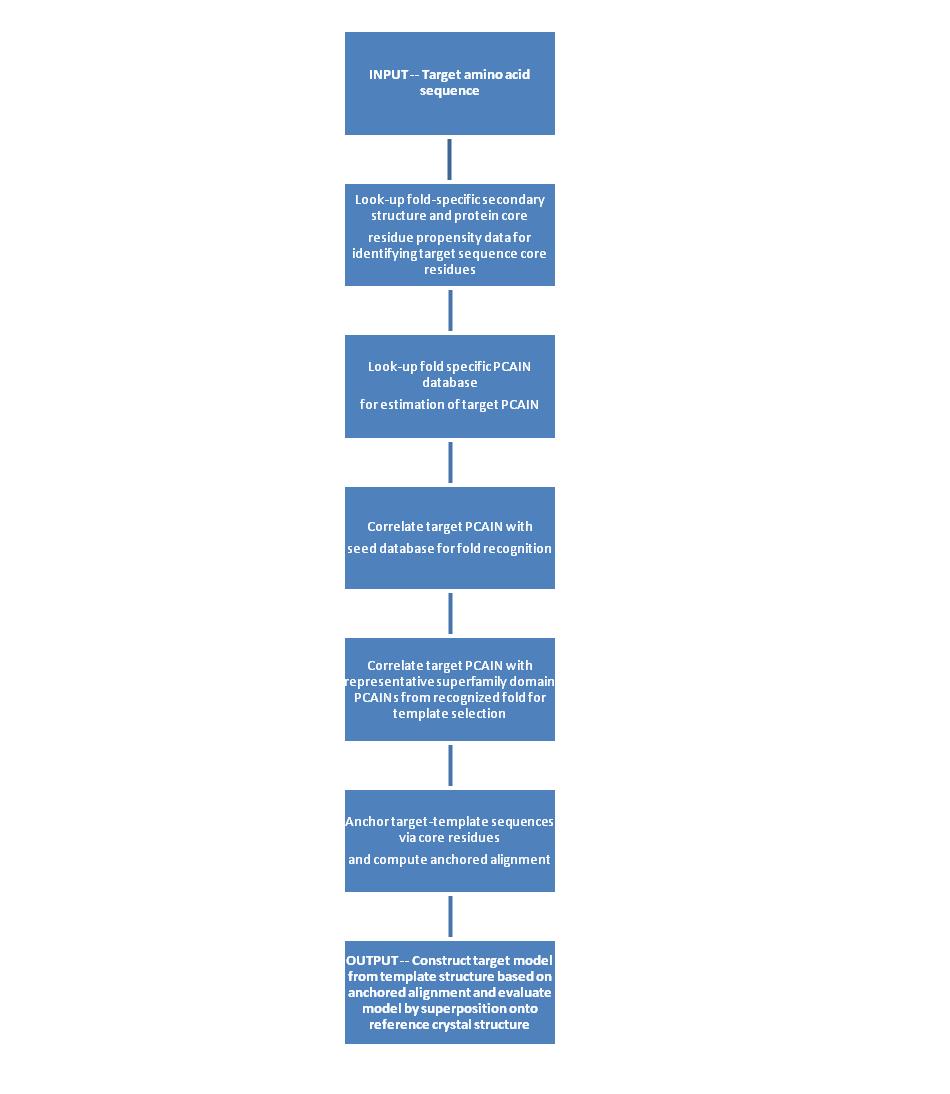

Supplement: Figure S6 — Flowchart governing PCAIN-based fold recognition of target sequence, template selection, anchored target-template alignment, and homology-based structure prediction. The detailed procedures associated with each step are described in the methods section. Briefly, a combination of secondary structure distribution and PCAIN scores from the key interaction positions was used to (i.) identify the fold of the target sequence, (ii.) compute the ideal template structure based on the closest functional homolog estimated from the superfamilies of the identified fold, (iii.) converge on the set of ‘anchor’ positions between the target and template sequences based on protein core amino acid frequencies to compute the optimal anchored target-template alignments, and (iv.) determine the target domain's 3-D structural coordinates from the anchored alignments with an automated homology modeling script. (0.05 MB JPG) [file pone.0009391.s006.jpg]

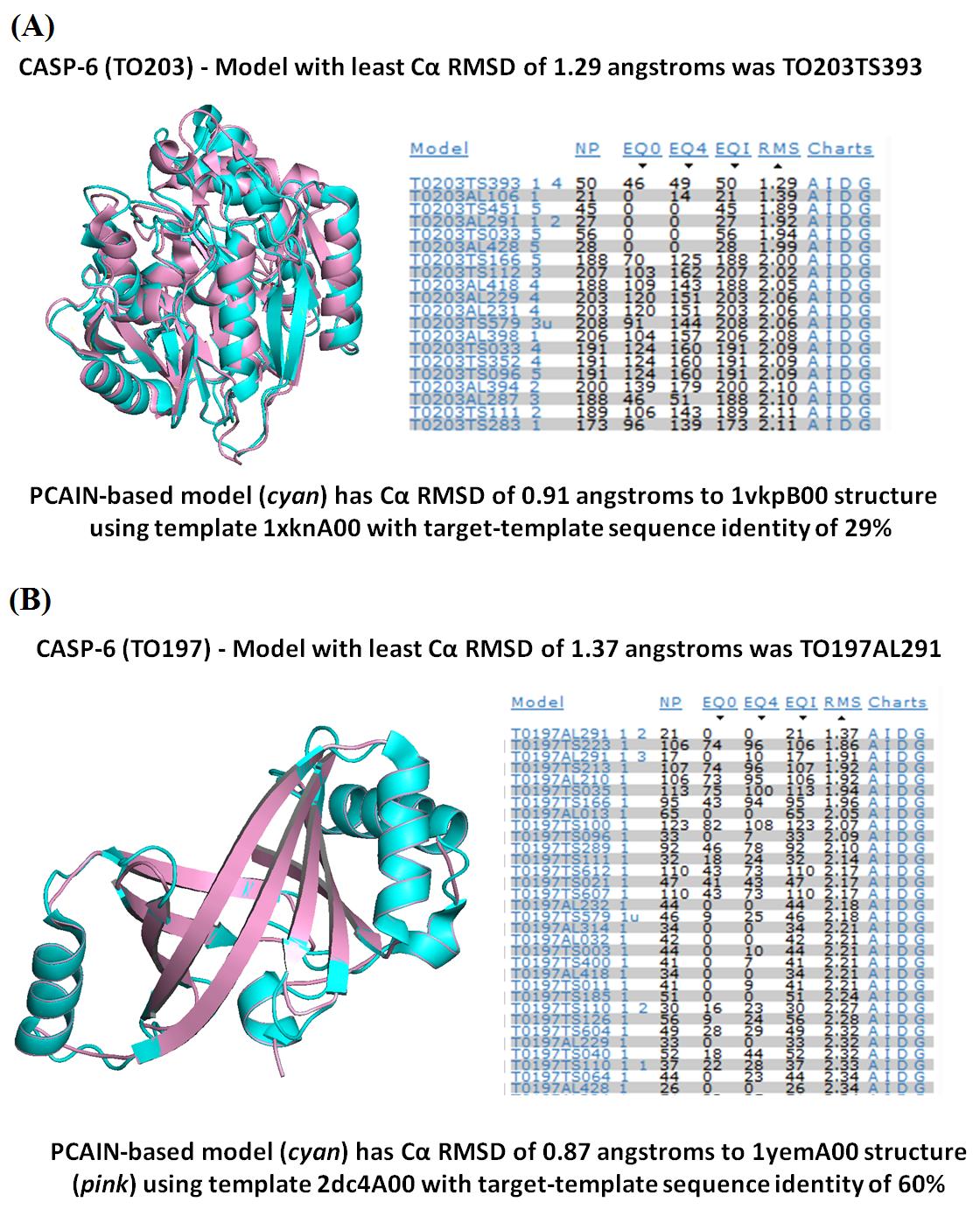

Supplement: Figure S7 — Superposition of structures predicted based on PCAIN methodology for CASP (Critical Assessment of Structure Prediction) target sequences (a.) TO203 and (b.) TO197, illustrates PCAIN-based structure prediction. PCAIN-based structures predicted (cyan) are superposed onto reference crystal structures (pink) for (a.) TOP203 and (b.) TO197 from CASP-6 with RMSDs of 0.91A (at 29% target-template sequence identity) and 0.87A (at 60% target-template sequence identity) respectively. The corresponding results of structure prediction accuracy from the CASP models shown as tables shows minimum RMSDs of 1.29A and 1.37A respectively. (0.26 MB JPG) [file pone.0009391.s007.jpg]

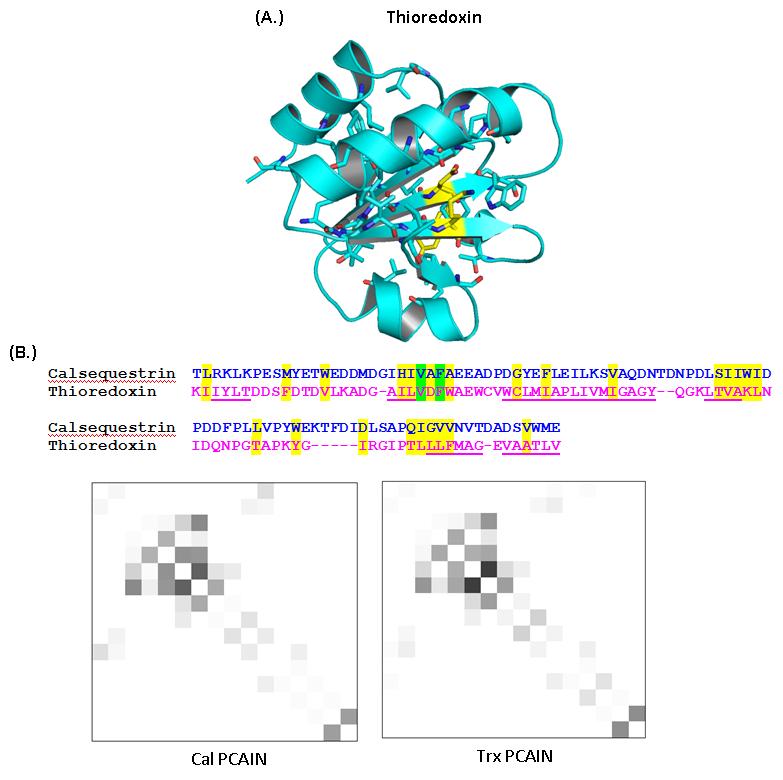

Supplement: Figure S8 — Defining protein cores and extracting their information with the PCAIN methodology. (A.) Polar and charged residues (yellow) are also part of the core of protein domain as identified by our method, as shown with E.coli thioredoxin (cyan) as an example. (B.) Only 7% identity (shaded green) is present in the sequence of residues that constitute the core of glutaredoxin and thioredoxin that adopt the same fold, whereas 93% of the core residues are different in identity (shaded yellow). However, the PCAINs of these two proteins are seen to have 98% correlation, over the PCMs that have only 41% correlation. This example further illustrates that the identity or hydrophobicity of residues are poor tools for extracting information from protein cores, whereas the PCAIN is optimal for extracting conserved information from protein cores. Similarly, very poor overlap is seen between residues used for CATH alignments (underlined) and the residues that contribute to the PCAIN, thus illustrating the novelty in determination of PCAIN residues. (0.07 MB JPG) [file pone.0009391.s008.jpg]
